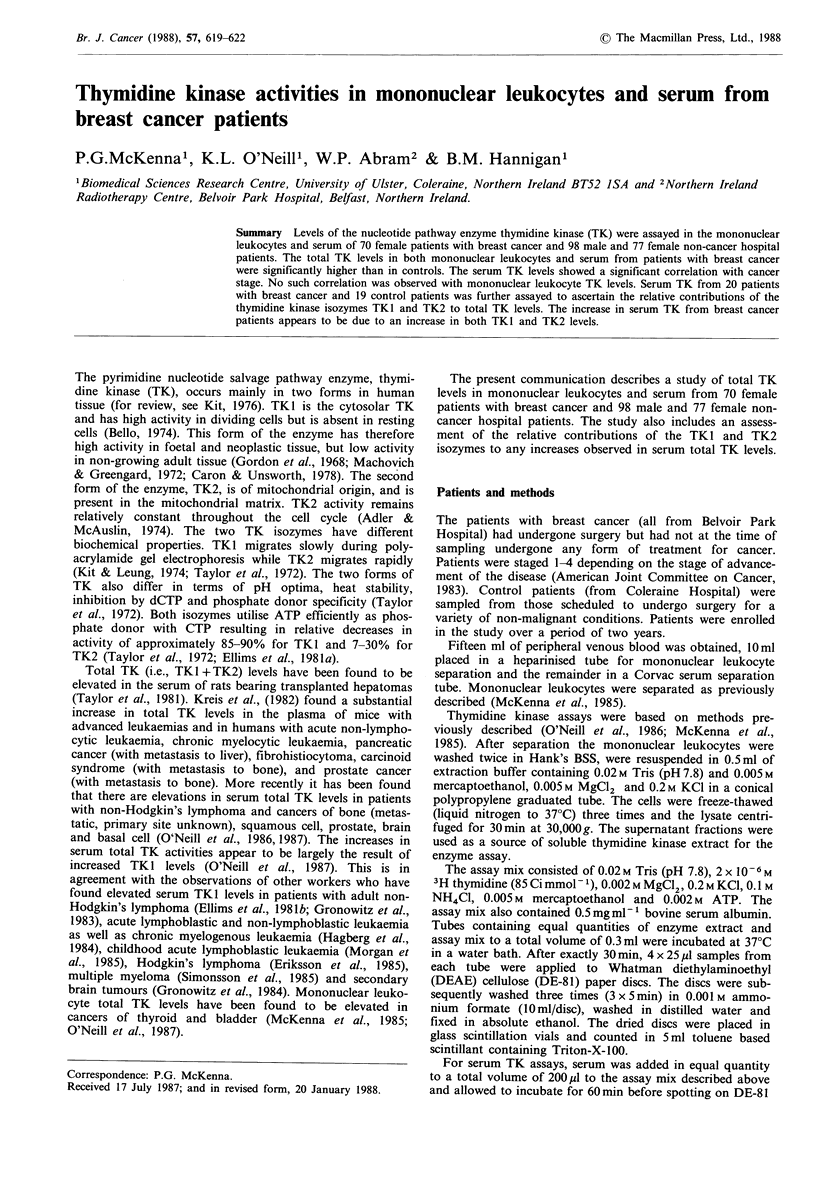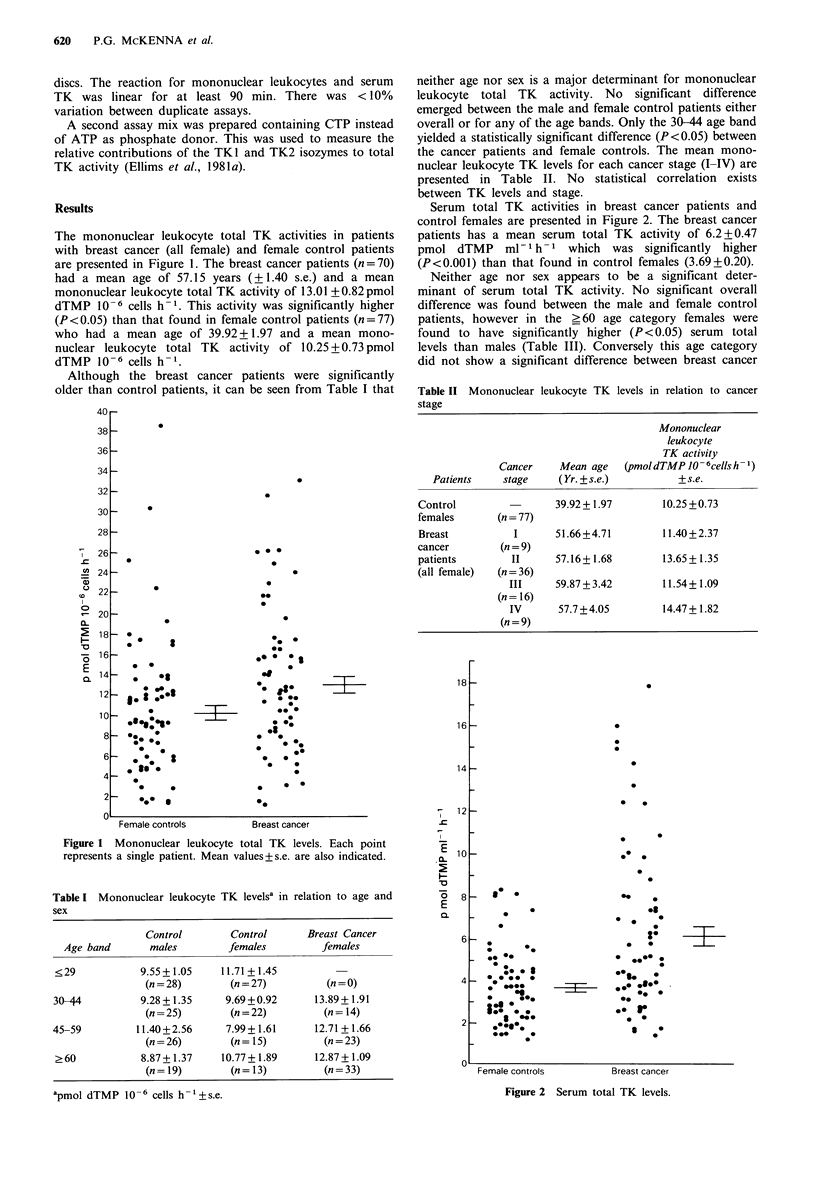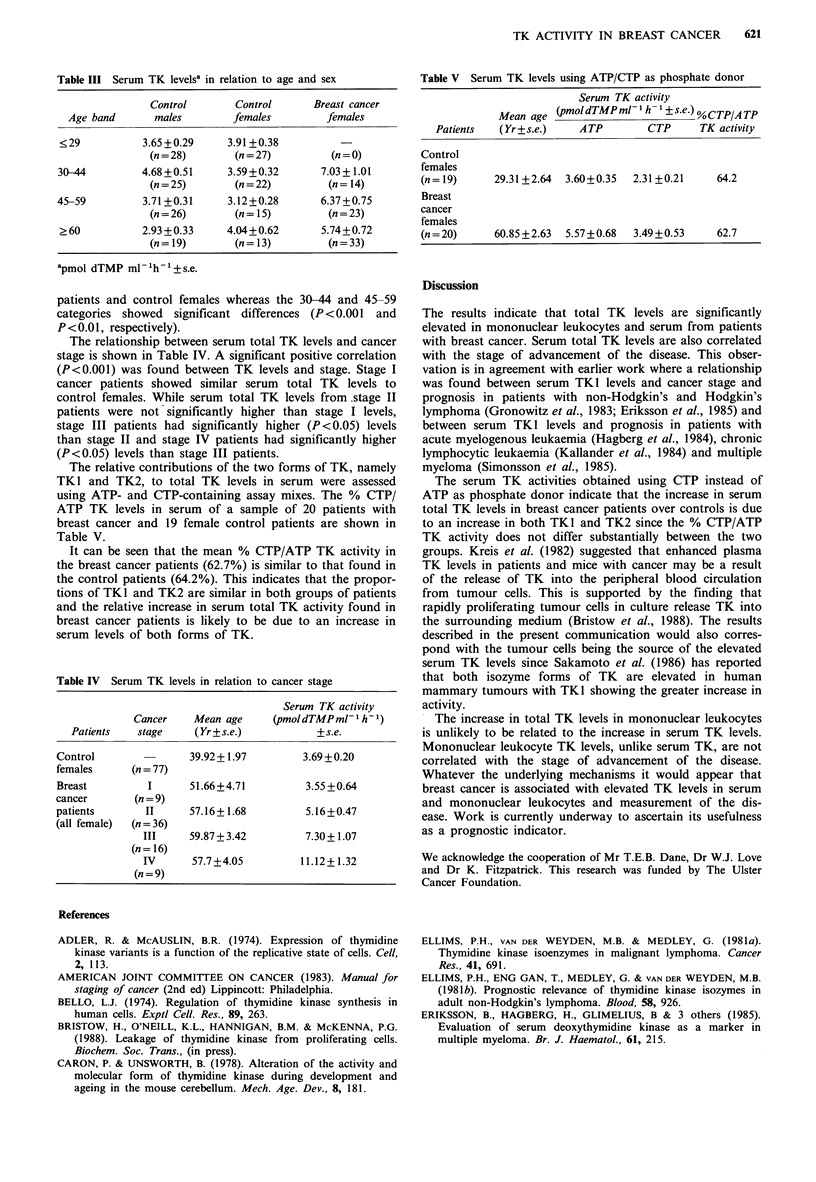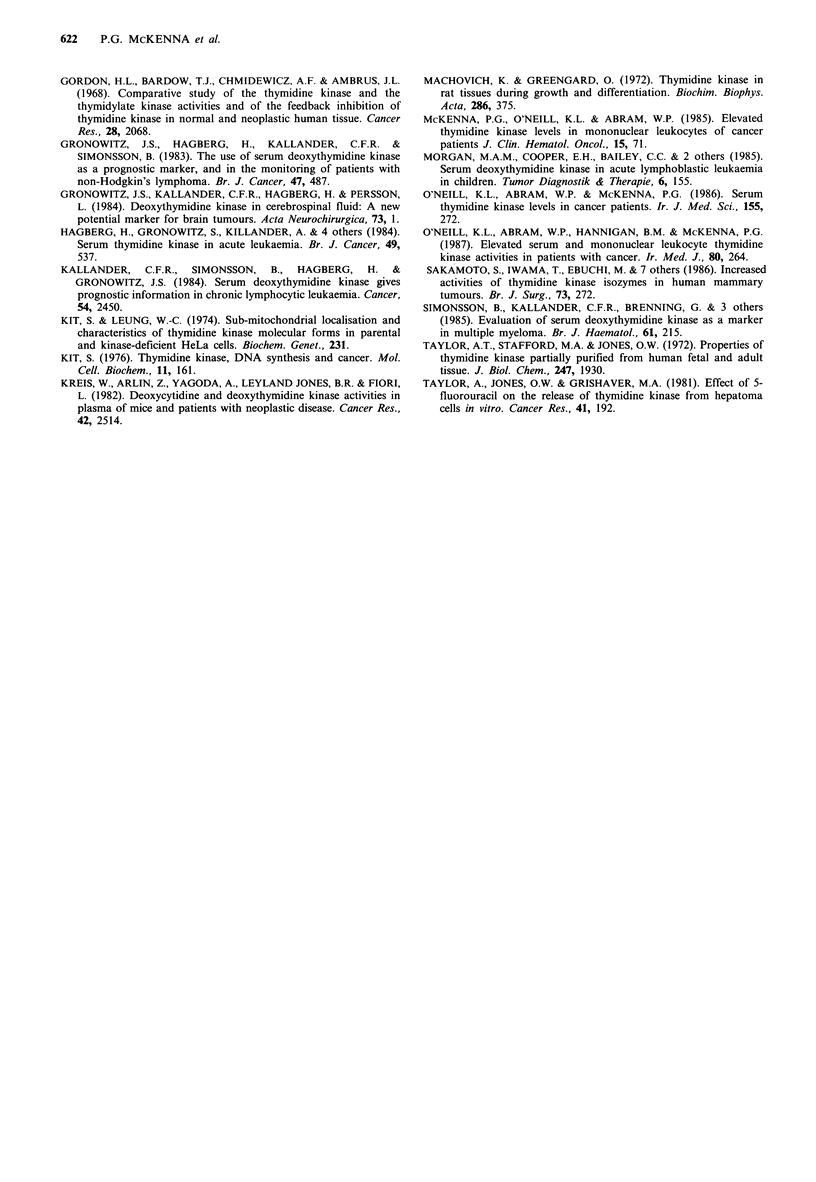# Thymidine kinase activities in mononuclear leukocytes and serum from breast cancer patients.

**DOI:** 10.1038/bjc.1988.141

**Published:** 1988-06

**Authors:** P. G. McKenna, K. L. O'Neill, W. P. Abram, B. M. Hannigan

**Affiliations:** Biomedical Sciences Research Centre, University of Ulster, Coleraine, Northern Ireland.

## Abstract

Levels of the nucleotide pathway enzyme thymidine kinase (TK) were assayed in the mononuclear leukocytes and serum of 70 female patients with breast cancer and 98 male and 77 female non-cancer hospital patients. The total TK levels in both mononuclear leukocytes and serum from patients with breast cancer were significantly higher than in controls. The serum TK levels showed a significant correlation with cancer stage. No such correlation was observed with mononuclear leukocyte TK levels. Serum TK from 20 patients with breast cancer and 19 control patients was further assayed to ascertain the relative contributions of the thymidine kinase isozymes TK1 and TK2 to total TK levels. The increase in serum TK from breast cancer patients appears to be due to an increase in both TK1 and TK2 levels.


Br. J. Cancer (1988), 57, 619–622 © The Macmillan Press, Ltd., 1988

### Thymidine kinase activities in mononuclear leukocytes and serum from breast cancer patients

P.G.McKenna[1], K.L. O'Neill[1], W.P. Abram[2] & B.M. Hannigan[1]

[1]*Biomedical Sciences Research Centre, University of Ulster, Coleraine, Northern Ireland BT52 1SA and* [2]*Northern Ireland Radiotherapy Centre, Belvoir Park Hospital, Belfast, Northern Ireland.*

**Summary** Levels of the nucleotide pathway enzyme thymidine kinase (TK) were assayed in the mononuclear leukocytes and serum of 70 female patients with breast cancer and 98 male and 77 female non-cancer hospital patients. The total TK levels in both mononuclear leukocytes and serum from patients with breast cancer were significantly higher than in controls. The serum TK levels showed a significant correlation with cancer stage. No such correlation was observed with mononuclear leukocyte TK levels. Serum TK from 20 patients with breast cancer and 19 control patients was further assayed to ascertain the relative contributions of the thymidine kinase isozymes TK1 and TK2 to total TK levels. The increase in serum TK from breast cancer patients appears to be due to an increase in both TK1 and TK2 levels.

The pyrimidine nucleotide salvage pathway enzyme, thymidine kinase (TK), occurs mainly in two forms in human tissue (for review, see Kit, 1976). TK1 is the cytosolar TK and has high activity in dividing cells but is absent in resting cells (Bello, 1974). This form of the enzyme has therefore high activity in foetal and neoplastic tissue, but low activity in non-growing adult tissue (Gordon *et al.*, 1968; Machovich & Greengard, 1972; Caron & Unsworth, 1978). The second form of the enzyme, TK2, is of mitochondrial origin, and is present in the mitochondrial matrix. TK2 activity remains relatively constant throughout the cell cycle (Adler & McAuslin, 1974). The two TK isozymes have different biochemical properties. TK1 migrates slowly during polyacrylamide gel electrophoresis while TK2 migrates rapidly (Kit & Leung, 1974; Taylor *et al.*, 1972). The two forms of TK also differ in terms of pH optima, heat stability, inhibition by dCTP and phosphate donor specificity (Taylor *et al.*, 1972). Both isozymes utilise ATP efficiently as phosphate donor with CTP resulting in relative decreases in activity of approximately 85–90% for TK1 and 7–30% for TK2 (Taylor *et al.*, 1972; Ellims *et al.*, 1981*a*).

Total TK (i.e., TK1 + TK2) levels have been found to be elevated in the serum of rats bearing transplanted hepatomas (Taylor *et al.*, 1981). Kreis *et al.*, (1982) found a substantial increase in total TK levels in the plasma of mice with advanced leukaemias and in humans with acute non-lymphocytic leukaemia, chronic myelocytic leukaemia, pancreatic cancer (with metastasis to liver), fibrohistiocytoma, carcinoid syndrome (with metastasis to bone), and prostate cancer (with metastasis to bone). More recently it has been found that there are elevations in serum total TK levels in patients with non-Hodgkin's lymphoma and cancers of bone (metastatic, primary site unknown), squamous cell, prostate, brain and basal cell (O'Neill *et al.*, 1986, 1987). The increases in serum total TK activities appear to be largely the result of increased TK1 levels (O'Neill *et al.*, 1987). This is in agreement with the observations of other workers who have found elevated serum TK1 levels in patients with adult non-Hodgkin's lymphoma (Ellims *et al.*, 1981*b*; Gronowitz *et al.*, 1983), acute lymphoblastic and non-lymphoblastic leukaemia as well as chronic myelogenous leukaemia (Hagberg *et al.*, 1984), childhood acute lymphoblastic leukaemia (Morgan *et al.*, 1985), Hodgkin's lymphoma (Eriksson *et al.*, 1985), multiple myeloma (Simonsson *et al.*, 1985) and secondary brain tumours (Gronowitz *et al.*, 1984). Mononuclear leukocyte total TK levels have been found to be elevated in cancers of thyroid and bladder (McKenna *et al.*, 1985; O'Neill *et al.*, 1987).

Correspondence: P.G. McKenna.
Received 17 July 1987; and in revised form, 20 January 1988.

The present communication describes a study of total TK levels in mononuclear leukocytes and serum from 70 female patients with breast cancer and 98 male and 77 female non-cancer hospital patients. The study also includes an assessment of the relative contributions of the TK1 and TK2 isozymes to any increases observed in serum total TK levels.

### Patients and methods

The patients with breast cancer (all from Belvoir Park Hospital) had undergone surgery but had not at the time of sampling undergone any form of treatment for cancer. Patients were staged 1–4 depending on the stage of advancement of the disease (American Joint Committee on Cancer, 1983). Control patients (from Coleraine Hospital) were sampled from those scheduled to undergo surgery for a variety of non-malignant conditions. Patients were enrolled in the study over a period of two years.

Fifteen ml of peripheral venous blood was obtained, 10 ml placed in a heparinised tube for mononuclear leukocyte separation and the remainder in a Corvac serum separation tube. Mononuclear leukocytes were separated as previously described (McKenna *et al.*, 1985).

Thymidine kinase assays were based on methods previously described (O'Neill *et al.*, 1986; McKenna *et al.*, 1985). After separation the mononuclear leukocytes were washed twice in Hank's BSS, were resuspended in 0.5 ml of extraction buffer containing 0.02 M Tris (pH 7.8) and 0.005 M mercaptoethanol, 0.005 M $MgCl_2$ and 0.2 M KCl in a conical polypropylene graduated tube. The cells were freeze-thawed (liquid nitrogen to 37°C) three times and the lysate centrifuged for 30 min at 30,000 *g*. The supernatant fractions were used as a source of soluble thymidine kinase extract for the enzyme assay.

The assay mix consisted of 0.02 M Tris (pH 7.8), $2 \times 10^{-6}$ M $^3H$ thymidine (85 Ci mmol$^{-1}$), 0.002 M $MgCl_2$, 0.2 M KCl, 0.1 M $NH_4Cl$, 0.005 M mercaptoethanol and 0.002 M ATP. The assay mix also contained 0.5 mg ml$^{-1}$ bovine serum albumin. Tubes containing equal quantities of enzyme extract and assay mix to a total volume of 0.3 ml were incubated at 37°C in a water bath. After exactly 30 min, $4 \times 25\,\mu l$ samples from each tube were applied to Whatman diethylaminoethyl (DEAE) cellulose (DE-81) paper discs. The discs were subsequently washed three times ($3 \times 5$ min) in 0.001 M ammonium formate (10 ml/disc), washed in distilled water and fixed in absolute ethanol. The dried discs were placed in glass scintillation vials and counted in 5 ml toluene based scintillant containing Triton-X-100.

For serum TK assays, serum was added in equal quantity to a total volume of 200 $\mu l$ to the assay mix described above and allowed to incubate for 60 min before spotting on DE-81

discs. The reaction for mononuclear leukocytes and serum TK was linear for at least 90 min. There was <10% variation between duplicate assays.

A second assay mix was prepared containing CTP instead of ATP as phosphate donor. This was used to measure the relative contributions of the TK1 and TK2 isozymes to total TK activity (Ellims *et al.*, 1981*a*).

### Results

The mononuclear leukocyte total TK activities in patients with breast cancer (all female) and female control patients are presented in Figure 1. The breast cancer patients ($n=70$) had a mean age of 57.15 years ($\pm 1.40$ s.e.) and a mean mononuclear leukocyte total TK activity of $13.01 \pm 0.82$ pmol dTMP $10^{-6}$ cells $h^{-1}$. This activity was significantly higher ($P<0.05$) than that found in female control patients ($n=77$) who had a mean age of $39.92 \pm 1.97$ and a mean mononuclear leukocyte total TK activity of $10.25 \pm 0.73$ pmol dTMP $10^{-6}$ cells $h^{-1}$.

Although the breast cancer patients were significantly older than control patients, it can be seen from Table I that neither age nor sex is a major determinant for mononuclear leukocyte total TK activity. No significant difference emerged between the male and female control patients either overall or for any of the age bands. Only the 30–44 age band yielded a statistically significant difference ($P<0.05$) between the cancer patients and female controls. The mean mononuclear leukocyte TK levels for each cancer stage (I–IV) are presented in Table II. No statistical correlation exists between TK levels and stage.

Serum total TK activities in breast cancer patients and control females are presented in Figure 2. The breast cancer patients has a mean serum total TK activity of $6.2 \pm 0.47$ pmol dTMP $ml^{-1} h^{-1}$ which was significantly higher ($P<0.001$) than that found in control females ($3.69 \pm 0.20$).

Neither age nor sex appears to be a significant determinant of serum total TK activity. No significant overall difference was found between the male and female control patients, however in the $\geq 60$ age category females were found to have significantly higher ($P<0.05$) serum total levels than males (Table III). Conversely this age category did not show a significant difference between breast cancer

**Figure 1** Mononuclear leukocyte total TK levels. Each point represents a single patient. Mean values ± s.e. are also indicated.

**Table I** Mononuclear leukocyte TK levels[a] in relation to age and sex

| *Age band* | *Control males* | *Control females* | *Breast Cancer females* |
|---|---|---|---|
| ≤29 | 9.55±1.05 ($n=28$) | 11.71±1.45 ($n=27$) | — ($n=0$) |
| 30–44 | 9.28±1.35 ($n=25$) | 9.69±0.92 ($n=22$) | 13.89±1.91 ($n=14$) |
| 45–59 | 11.40±2.56 ($n=26$) | 7.99±1.61 ($n=15$) | 12.71±1.66 ($n=23$) |
| ≥60 | 8.87±1.37 ($n=19$) | 10.77±1.89 ($n=13$) | 12.87±1.09 ($n=33$) |

[a]pmol dTMP $10^{-6}$ cells $h^{-1} \pm$ s.e.

**Table II** Mononuclear leukocyte TK levels in relation to cancer stage

| *Patients* | *Cancer stage* | *Mean age (Yr. ± s.e.)* | *Mononuclear leukocyte TK activity (pmol dTMP $10^{-6}$ cells $h^{-1}$) ± s.e.* |
|---|---|---|---|
| Control females | — ($n=77$) | 39.92±1.97 | 10.25±0.73 |
| Breast cancer patients (all female) | I ($n=9$) | 51.66±4.71 | 11.40±2.37 |
| | II ($n=36$) | 57.16±1.68 | 13.65±1.35 |
| | III ($n=16$) | 59.87±3.42 | 11.54±1.09 |
| | IV ($n=9$) | 57.7±4.05 | 14.47±1.82 |

**Figure 2** Serum total TK levels.

**Table III** Serum TK levels[a] in relation to age and sex

| Age band | Control males | Control females | Breast cancer females |
|---|---|---|---|
| ≤29 | 3.65±0.29 ($n$=28) | 3.91±0.38 ($n$=27) | — ($n$=0) |
| 30–44 | 4.68±0.51 ($n$=25) | 3.59±0.32 ($n$=22) | 7.03±1.01 ($n$=14) |
| 45–59 | 3.71±0.31 ($n$=26) | 3.12±0.28 ($n$=15) | 6.37±0.75 ($n$=23) |
| ≥60 | 2.93±0.33 ($n$=19) | 4.04±0.62 ($n$=13) | 5.74±0.72 ($n$=33) |

[a]pmol dTMP $ml^{-1}h^{-1}$±s.e.

patients and control females whereas the 30–44 and 45–59 categories showed significant differences ($P<0.001$ and $P<0.01$, respectively).

The relationship between serum total TK levels and cancer stage is shown in Table IV. A significant positive correlation ($P<0.001$) was found between TK levels and stage. Stage I cancer patients showed similar serum total TK levels to control females. While serum total TK levels from stage II patients were not significantly higher than stage I levels, stage III patients had significantly higher ($P<0.05$) levels than stage II and stage IV patients had significantly higher ($P<0.05$) levels than stage III patients.

The relative contributions of the two forms of TK, namely TK1 and TK2, to total TK levels in serum were assessed using ATP- and CTP-containing assay mixes. The % CTP/ATP TK levels in serum of a sample of 20 patients with breast cancer and 19 female control patients are shown in Table V.

It can be seen that the mean % CTP/ATP TK activity in the breast cancer patients (62.7%) is similar to that found in the control patients (64.2%). This indicates that the proportions of TK1 and TK2 are similar in both groups of patients and the relative increase in serum total TK activity found in breast cancer patients is likely to be due to an increase in serum levels of both forms of TK.

**Table IV** Serum TK levels in relation to cancer stage

| Patients | Cancer stage | Mean age (*Yr*±*s.e.*) | Serum TK activity (*pmol dTMP* $ml^{-1}h^{-1}$) ±*s.e.* |
|---|---|---|---|
| Control females | — ($n$=77) | 39.92±1.97 | 3.69±0.20 |
| Breast cancer patients (all female) | I ($n$=9) | 51.66±4.71 | 3.55±0.64 |
| | II ($n$=36) | 57.16±1.68 | 5.16±0.47 |
| | III ($n$=16) | 59.87±3.42 | 7.30±1.07 |
| | IV ($n$=9) | 57.7±4.05 | 11.12±1.32 |

**Table V** Serum TK levels using ATP/CTP as phosphate donor

| Patients | Mean age (*Yr*±*s.e.*) | Serum TK activity (*pmol dTMP* $ml^{-1}h^{-1}$±*s.e.*) ATP | CTP | %CTP/ATP TK activity |
|---|---|---|---|---|
| Control females ($n$=19) | 29.31±2.64 | 3.60±0.35 | 2.31±0.21 | 64.2 |
| Breast cancer females ($n$=20) | 60.85±2.63 | 5.57±0.68 | 3.49±0.53 | 62.7 |

### Discussion

The results indicate that total TK levels are significantly elevated in mononuclear leukocytes and serum from patients with breast cancer. Serum total TK levels are also correlated with the stage of advancement of the disease. This observation is in agreement with earlier work where a relationship was found between serum TK1 levels and cancer stage and prognosis in patients with non-Hodgkin's and Hodgkin's lymphoma (Gronowitz *et al.*, 1983; Eriksson *et al.*, 1985) and between serum TK1 levels and prognosis in patients with acute myelogenous leukaemia (Hagberg *et al.*, 1984), chronic lymphocytic leukaemia (Kallander *et al.*, 1984) and multiple myeloma (Simonsson *et al.*, 1985).

The serum TK activities obtained using CTP instead of ATP as phosphate donor indicate that the increase in serum total TK levels in breast cancer patients over controls is due to an increase in both TK1 and TK2 since the % CTP/ATP TK activity does not differ substantially between the two groups. Kreis *et al.* (1982) suggested that enhanced plasma TK levels in patients and mice with cancer may be a result of the release of TK into the peripheral blood circulation from tumour cells. This is supported by the finding that rapidly proliferating tumour cells in culture release TK into the surrounding medium (Bristow *et al.*, 1988). The results described in the present communication would also correspond with the tumour cells being the source of the elevated serum TK levels since Sakamoto *et al.* (1986) has reported that both isozyme forms of TK are elevated in human mammary tumours with TK1 showing the greater increase in activity.

The increase in total TK levels in mononuclear leukocytes is unlikely to be related to the increase in serum TK levels. Mononuclear leukocyte TK levels, unlike serum TK, are not correlated with the stage of advancement of the disease. Whatever the underlying mechanisms it would appear that breast cancer is associated with elevated TK levels in serum and mononuclear leukocytes and measurement of the disease. Work is currently underway to ascertain its usefulness as a prognostic indicator.

We acknowledge the cooperation of Mr T.E.B. Dane, Dr W.J. Love and Dr K. Fitzpatrick. This research was funded by The Ulster Cancer Foundation.

### References

ADLER, R. & MCAUSLIN, B.R. (1974). Expression of thymidine kinase variants is a function of the replicative state of cells. *Cell*, **2**, 113.

AMERICAN JOINT COMMITTEE ON CANCER (1983). *Manual for staging of cancer* (2nd ed) Lippincott: Philadelphia.

BELLO, L.J. (1974). Regulation of thymidine kinase synthesis in human cells. *Exptl Cell. Res.*, **89**, 263.

BRISTOW, H., O'NEILL, K.L., HANNIGAN, B.M. & MCKENNA, P.G. (1988). Leakage of thymidine kinase from proliferating cells. *Biochem. Soc. Trans.*, (in press).

CARON, P. & UNSWORTH, B. (1978). Alteration of the activity and molecular form of thymidine kinase during development and ageing in the mouse cerebellum. *Mech. Age. Dev.*, **8**, 181.

ELLIMS, P.H., VAN DER WEYDEN, M.B. & MEDLEY, G. (1981*a*). Thymidine kinase isoenzymes in malignant lymphoma. *Cancer Res.*, **41**, 691.

ELLIMS, P.H., ENG GAN, T., MEDLEY, G. & VAN DER WEYDEN, M.B. (1981*b*). Prognostic relevance of thymidine kinase isozymes in adult non-Hodgkin's lymphoma. *Blood*, **58**, 926.

ERIKSSON, B., HAGBERG, H., GLIMELIUS, B & 3 others (1985). Evaluation of serum deoxythymidine kinase as a marker in multiple myeloma. *Br. J. Haematol.*, **61**, 215.

GORDON, H.L., BARDOW, T.J., CHMIDEWICZ, A.F. & AMBRUS, J.L. (1968). Comparative study of the thymidine kinase and the thymidylate kinase activities and of the feedback inhibition of thymidine kinase in normal and neoplastic human tissue. *Cancer Res.*, **28**, 2068.

GRONOWITZ, J.S., HAGBERG, H., KALLANDER, C.F.R. & SIMONSSON, B. (1983). The use of serum deoxythymidine kinase as a prognostic marker, and in the monitoring of patients with non-Hodgkin's lymphoma. *Br. J. Cancer*, **47**, 487.

GRONOWITZ, J.S., KALLANDER, C.F.R., HAGBERG, H. & PERSSON, L. (1984). Deoxythymidine kinase in cerebrospinal fluid: A new potential marker for brain tumours. *Acta Neurochirurgica*, **73**, 1.

HAGBERG, H., GRONOWITZ, S., KILLANDER, A. & 4 others (1984). Serum thymidine kinase in acute leukaemia. *Br. J. Cancer*, **49**, 537.

KALLANDER, C.F.R., SIMONSSON, B., HAGBERG, H. & GRONOWITZ, J.S. (1984). Serum deoxythymidine kinase gives prognostic information in chronic lymphocytic leukaemia. *Cancer*, **54**, 2450.

KIT, S. & LEUNG, W.-C. (1974). Sub-mitochondrial localisation and characteristics of thymidine kinase molecular forms in parental and kinase-deficient HeLa cells. *Biochem. Genet.*, **231**.

KIT, S. (1976). Thymidine kinase, DNA synthesis and cancer. *Mol. Cell. Biochem.*, **11**, 161.

KREIS, W., ARLIN, Z., YAGODA, A., LEYLAND JONES, B.R. & FIORI, L. (1982). Deoxycytidine and deoxythymidine kinase activities in plasma of mice and patients with neoplastic disease. *Cancer Res.*, **42**, 2514.

MACHOVICH, K. & GREENGARD, O. (1972). Thymidine kinase in rat tissues during growth and differentiation. *Biochim. Biophys. Acta*, **286**, 375.

McKENNA, P.G., O'NEILL, K.L. & ABRAM, W.P. (1985). Elevated thymidine kinase levels in mononuclear leukocytes of cancer patients *J. Clin. Hematol. Oncol.*, **15**, 71.

MORGAN, M.A.M., COOPER, E.H., BAILEY, C.C. & 2 others (1985). Serum deoxythymidine kinase in acute lymphoblastic leukaemia in children. *Tumor Diagnostik & Therapie*, **6**, 155.

O'NEILL, K.L., ABRAM, W.P. & McKENNA, P.G. (1986). Serum thymidine kinase levels in cancer patients. *Ir. J. Med. Sci.*, **155**, 272.

O'NEILL, K.L., ABRAM, W.P., HANNIGAN, B.M. & McKENNA, P.G. (1987). Elevated serum and mononuclear leukocyte thymidine kinase activities in patients with cancer. *Ir. Med. J.*, **80**, 264.

SAKAMOTO, S., IWAMA, T., EBUCHI, M. & 7 others (1986). Increased activities of thymidine kinase isozymes in human mammary tumours. *Br. J. Surg.*, **73**, 272.

SIMONSSON, B., KALLANDER, C.F.R., BRENNING, G. & 3 others (1985). Evaluation of serum deoxythymidine kinase as a marker in multiple myeloma. *Br. J. Haematol.*, **61**, 215.

TAYLOR, A.T., STAFFORD, M.A. & JONES, O.W. (1972). Properties of thymidine kinase partially purified from human fetal and adult tissue. *J. Biol. Chem.*, **247**, 1930.

TAYLOR, A., JONES, O.W. & GRISHAVER, M.A. (1981). Effect of 5-fluorouracil on the release of thymidine kinase from hepatoma cells *in vitro*. *Cancer Res.*, **41**, 192.